# Monitoring and enhancing the co-operation of IoT network rhrough scheduling function based punishment reward strategy

**DOI:** 10.1371/journal.pone.0309123

**Published:** 2024-09-19

**Authors:** Abdur Rashid Sangi, Bingqian Li, Satish Anamalamudi, Anil Carie

**Affiliations:** 1 Wenzhou-Kean University, Wenzhou, P. R. China; 2 Kean University, Union, NJ, United States of America; 3 Soochow University, Suzhou, P. R. China; 4 SRM University-AP, Amaravati, India; Saint Louis University, UNITED STATES OF AMERICA

## Abstract

The Internet of Things (IoT) has revolutionized the connectivity of physical devices, leading to an exponential increase in multimedia wireless traffic and creating substantial demand for radio spectrum. Given the inherent scarcity of available spectrum, Cognitive Radio (CR)-assisted IoT emerges as a promising solution to optimize spectrum utilization through cooperation between cognitive and IoT nodes. Unlicensed IoT nodes can opportunistically access licensed spectrum bands without causing interference to licensed users. However, energy constraints may lead to reduced cooperation from IoT nodes during the search for vacant channels, as they aim to conserve battery life. To address this issue, we propose a Punishment-reward-based Cooperative Sensing and Data Forwarding (PR-CSDF) approach for IoT data transmission. Our method involves two key steps: (1) distributing sensing tasks among IoT nodes and (2) enhancing cooperation through a reward and punishment strategy. Evaluation results demonstrate that both secondary users (SUs) and IoT nodes achieve significant utility gains with the proposed mechanism, providing strong incentives for cooperative behaviour.

## 1 Introduction

**Fig 1:**
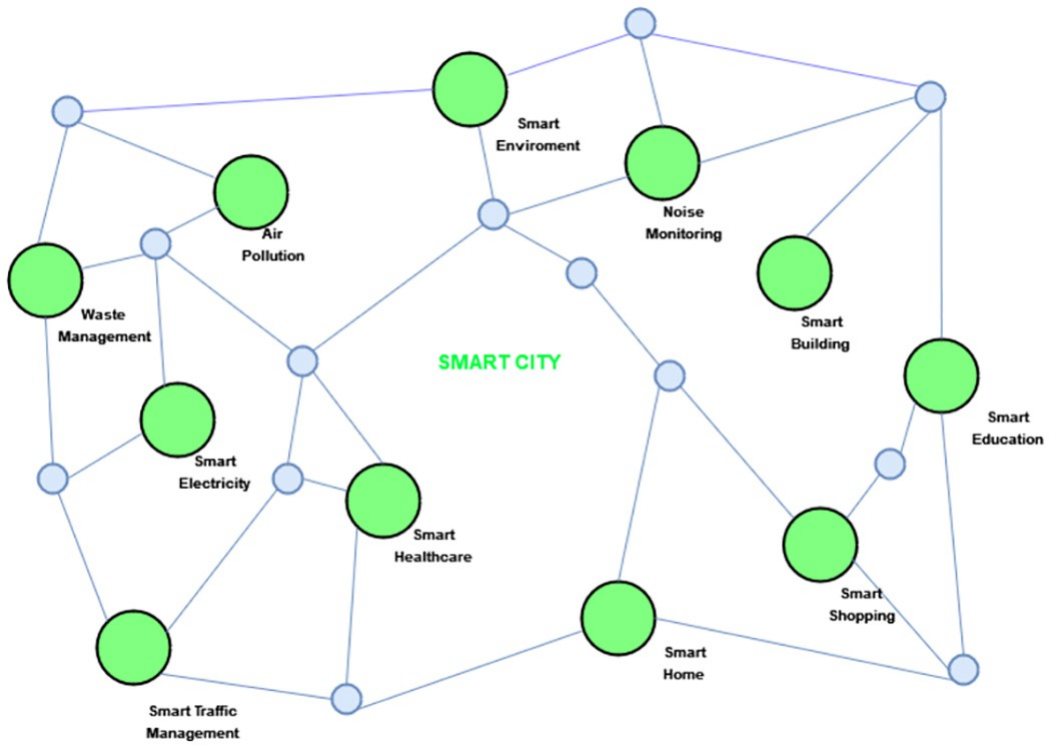
Smart city with different smart IoT nodes.

The rapid advancements in wireless communication have set off new paradigms in wireless networking. Currently, the research community is focusing on 5G to provide mobile broadband services in wireless networks for providing internet connectivity from every electronic device to IoT nodes [1]. IoT, which is a key feature of the 5G wireless network that provides internet connectivity to physical devices, has been able to realize the requirements of a smart city. The smart city mission is to deploy IoT nodes in the interested regions like hospitals, transportation applications, security applications, and power grids for monitoring and collecting data as well as for efficient management [2]. The focus is on addressing the challenges posed by the massive amount of data generated by IoT devices [3]. The integrated communication and information technologies are used in managing the city as shown in Fig 1.

Nevertheless, the use of IoT technology in realizing smart cities comes along with a set of challenges. According to [4], the number of devices that will be connected to the internet by 2025 will be 75.44 billion worldwide. Because of it, there will be a huge increase in multimedia wireless traffic which leads to an increase in the demand for the required natural available spectrum that causes spectrum shortage. The primary reason for the spectrum scarcity is due to fixed static channel allocation strategy and increased demand for spectrum access in the latest wireless technologies [5]. To handle the spectrum scarcity problem, the existing spectrum bands (licensed and unlicensed) should be efficiently utilized. Cognitive radio-assisted wireless devices can access the licensed channel opportunistically to alleviate the existing spectrum scarcity problem by allowing unlicensed users to access the licensed channel [6]. In other words, when the licensed user is idle at a particular geographical location with respect to time then the cognitive user can access the channel without causing interference to the licensed primary user.

### 1.1 Motivation

In cognitive radio-assisted IoT networks, two important aspects have to consider: first is task allocation and the second is cooperation among cognitive IoT nodes. Cognitive nodes need to distribute the sensing task among the neighbouring sensing nodes for distributed communication. Numerous task allocation approaches have been studied in state-of-the-art traditional wireless networks [7]. However, the existing approaches cannot be directly applied to IoT networks due to constrained node resources and special wireless scenarios. The design of the task allocation approach for cognitive IoT is ongoing research [8]. Subsequently, how mutual cooperation can be achieved in between cognitive node and IoT nodes has to be explored to have interference-free communication. Recently, cooperation among the wireless nodes to exchange both the application data and spectrum-related information has been explored in [9]. In [10], the performance of the IoT network is being enhanced by allowing the primary user’s licensed channel access to an unlicensed user with the condition that the unlicensed user cooperates by acting as a relay for edge licensed nodes. Since the network adapters are more and more modifiable, it allows selfish nodes to tinker with the wireless interface and maximizes their own benefit. This leads to natural and important design issues like: *how to deal with selfish nodes? and what if the majority of nodes are selfish?* Thus, this paper works on two important issues namely 1. Nodes in Cognitive IoT need to distribute the sensing task among the neighbour sensing nodes with unknown nature and 2. To deal with the selfish nodes within the network.

### 1.2 Limitations in existing work

With respect to a task allocation, studies focus to select a group of nodes to complete a global application cooperatively by using genetic algorithm (GA) [11] or partial swarm optimization (PSO) [12]. Other models are proposed by modifying PSO, GA where the models suppose to know all the network parameters in advance. However, in a network with autonomous nodes whose behaviour is based on their current state, it is difficult to solve this requirement in a real-world IoT scenario.

State-of-the-art research has shown that selfish nodes seriously deter the performance of the network [13–15]. In general, there are three ways that have been used to deal with selfishness in ad-hoc networks in the literature a) precautions of selfish nodes [16]; b) detection of selfish nodes [17, 18]; c) avoidance of selfish nodes [19]. Each of these approaches has its own unique advantages and disadvantages. Lin *et al*. [20] proposed CONFIDANT algorithm which acquires reputation value and excludes network method to penalize non-cooperative nodes. In [21], security mechanisms have been implemented to stop nodes from tampering with vital information. Most of the existing research solves the selfishness issue in IoT nodes by using model-based data forwarding scenarios i.e., a node is considered to be selfish in case it does not forward data to the neighbour node. Otherwise, it is considered as the cooperating node. In [22], author addresses the limitations of IPv6 RPL in IoT networks’ extreme conditions and introduces Dynamic-RPL. With minimal changes to RPL, Dynamic-RPL ensures high network performance, adaptability to adverse conditions, and efficient topology management. Different from previous studies, in this work, we address the selfishness in IoT nodes by analyzing the node’s unwillingness to cooperate with the cognitive node in identifying the vacant licensed bands.

It is noteworthy that selfishness in cognitive radio-assisted IoT scenarios is different from selfishness in the traditional wireless network in terms of channel allocation. In a traditional network, when node acts selfishly, it can save its energy and prolong the battery lifetime. But in the case of selfishness in cognitive radio wireless networks, there can be an interference with primary users(PU) by not co-operating the data transmission in an opportunistic licensed channel [23]. Even though literature work addresses the issue of selfish nodes in a wireless network, little work has been done in terms of setting specific rules to regulate in terms of energy conservation and truthfulness. Thus, how to identify the selfish node activity which results in increasing the lifetime of IoT nodes will be addressed in this paper.

### 1.3 Proposed solution

**Fig 2:**
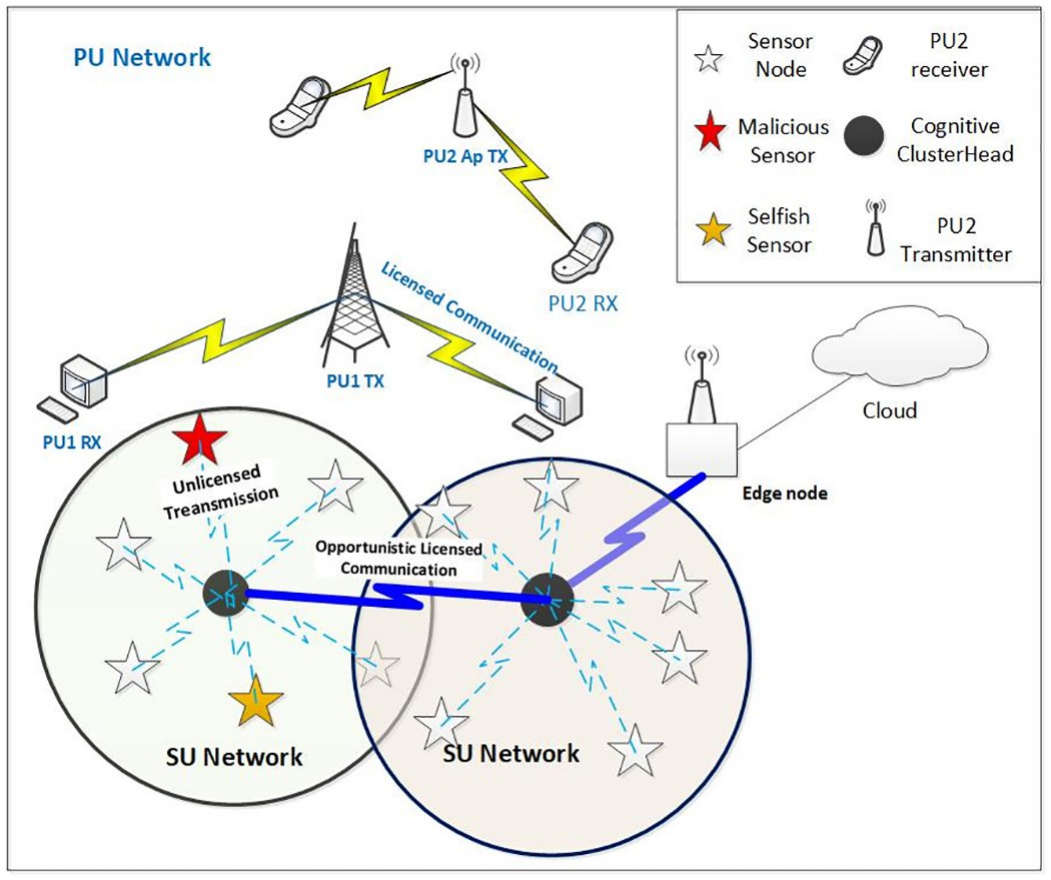
System architecture: Cognitive-IoT network with selfish and malicious nodes.

From Fig 2, cognitive nodes are going to act as cluster heads for the IoT nodes. Cognitive nodes distribute the sensing task to the neighbour IoT nodes while the participating IoT nodes can contribute to the sensing licensed channel. To address task allocation and selfish node detection, this paper proposes a game theory-based cooperative model for IoT nodes to participate in sensing tasks initiated by cognitive nodes. Firstly, we use the auction model to assign tasks and assign a reward to IoT nodes. Subsequently, for enhancing cooperation, the cognitive node rewards selfless IoT nodes based on shapely value and punishes the selfish IoT nodes based on trust value. The key concepts of this paper are summarized as follows:

In this paper, we propose a cooperative-based model for users in fog and cloud computing to participate in tasks published by an application platform. In the formulation process, the participating users can contribute to the tasks, and the tasks will pay the users rewards. The reward to the users in this paper is defined to have a relationship with region density. The proposed idea is a two-step approach to deal with selfish nodes. In the first step, we use a prevention mechanism where the cognitive node scans the network frequently to discover the selfishness. In the second step, we use an avoidance mechanism that will promote cooperation among the IoT nodes using a reward and punishment strategy. Thus we are motivated to develop a mechanism for selfish nodes in the cognitive IoT network using a game-theoretic approach to improve the cooperation with constraints on energy. In this paper, we have two mechanisms namely 1) frequency of monitoring and 2) promotion of cooperation through punishment and reward [24]. The cooperative mechanism uses two partial and full monitoring to understand the behaviour of the nodes. The key concepts of this paper are summarized as follows:

(1) The players of the game are IoT nodes that enable to choose in between two strategies. One is to cooperate with the cognitive node and the other is to defect. Cooperation and defection have an impact on the quality of the licensed channel in terms of throughput.
(2) Auction mechanism is used by a cognitive node for the distribution of sensing tasks among the IoT nodes.
(3) Monitoring the behaviour of nodes requires energy, thus monitoring nodes need to know optimal monitoring frequency to save energy and identify the nodes’ behaviour.
(4) To promote cooperation in the network and reduce selfish nodes, the identification of selfish nodes is necessary. Partial and full monitoring is used to punish selfish nodes and reward cooperative nodes.
(5) Proposed ‘Malicious node metrics’ to identify and reduce the occurrence of Malicious nodes.

The rest of the paper is organized as follows: Section 2 introduces the theoretical basis and specific process of the PR-CSDF mechanism. Section 3 provides the pseudocode of proposed algorithms in the paper. The performance of the proposed work is evaluated in Section 4 and Finally, Section 5 concludes the paper with outlines of our future work.

## 2 System model

### 2.1 Problem scenario

The network topology consists of the secondary network consisting of IoT nodes, cognitive nodes(CNs) co-existing with the primary network with primary users(PUs) as shown in Fig 2. Cognitive nodes are allowed to access the PU licensed channel when it is not used by primary users(overlay access) or with limited power without causing interference to the ongoing PU transmissions(underlay access). However, cognitive nodes need to sense the licensed channel to identify spectrum opportunities and avoid interference with PUs. Sometimes CN cannot detect the presence of primary transmitters due to hidden terminal problems. Thus, cooperative strategies are preferred for sensing primary user transmissions. Here, the CN node distributes the sensing tasks to the nearby IoT nodes and rewards them with the opportunity to transmit in a licensed channel. This reward is the motivation for IoT nodes, as unlicensed channels are not reliable for data transmissions and IoT nodes need to do multiple re-transmission. Thus, cooperation gives benefits to both cognitive nodes and IoT nodes, notation used are given in Table 1.

**Table 1:** Basic notations.

*P*_*o*_	The probablity of retransmissions
*m*_*l*_	The number of retransmissions in licensed channel
*m*_*ul*_	The number of retransmissions in unlicensed channel
*b*_*i*_	The bid of node i
*E*^*TX*^(*L*, *d*)	Transmit energy consumed
*v*_*i*_	Private value of node i
$t_{d_{i}}$	The direct trust value of node i
*N*_*c*_	The amount of successful cooperations
*N*_*d*_	The amount of selfish behaviors
$t_{in_{ij}}$	The indirect trust value from node i to node j
*fre*	The frequency of monitoring
*R*	Reserve price
*φ*_*i*_(*l*)	The reward of node i
*Similarity*_*i*,*j*_	The similarity of value between node i and j

### 2.2 Overview of proposed solution

**Fig 3:**
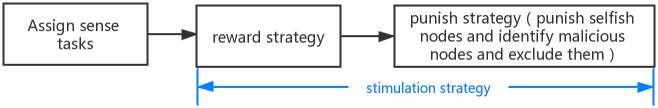
System process.

In Fig 3, to capture the interaction between CN and IoT nodes, we have used multi-auction where CN is the auctioneer and IoT nodes are bidders (2.4.2). The cognitive node which is *auctioneer* broadcasts the sensing task to the neighbouring IoT nodes. Rational IoT nodes which are *bidders* send the *bid* *B* = *B*_1_, *B*_2_, *B*_3_, *B*_4_, …., *B*_*n*_, *B*_*i*_ denotes the amount of sensing time allocated to cognitive node. Every IoT node sends its bid values to obtain rewards from CN with the promise of its contribution. More specifically, the sensing time they are willing to offer. By analysing the payoff of CN and IoT nodes, the upper bound and lower bound of feasible bids are calculated by energy consumption in the licensed and unlicensed channels. In such a way, tasks will be assigned to selfless nodes in most cases. But, selfish IoT nodes in the network impact the system performance.

A game theory model based on the IoT node’s behaviors(i.e, selfish or selfless) and the corresponding payoff is built. Using this model, we understand that both reward and punishment strategies need to be applied. It provides the direction for the next modeling. Meanwhile, the optimal monitoring frequency formula is presented with the analysis process of the payoff matrix. This formula can help CN to avoid unnecessary energy consumption while dynamic frequency is employed in every round.

The cooperation process which includes reward and punishment mechanisms has three aspects. At First, indirect trust values will put forward to adjust the frequency. Secondly, nodes are rewarded with additional sensing using shapely values. In the end, over a period of time, CN analyzes the data sent by its own monitoring data, i.e., its direct trust value, and punishes the selfish IoT nodes. Trust value is a credit record for previous behaviors. CN decides whether or not to punish and which degree of punishment will be chosen by direct trust value and change the frequency value by indirect trust value. To deal with selfish IoT nodes, a reward and punishment strategy is applied. Here, CN monitors selfish IoT nodes using the optimal frequency formula in order to avoid unnecessary energy consumption. A game theory model based on the behaviors and the corresponding payoff is built to find the best strategy to deal with selfish IoT nodes. Both reward and punishment should be applied according to the analysis result. Furthermore, the optimal frequency formula is given in the meantime.

In the course of the system’s operation, it may be more likely to develop malicious nodes as the number of selfish nodes increase. The exert of malicious nodes identification will reduce the benefit to transform into malicious nodes, and thus, reduce the likelihood of the appearance of malicious nodes and affecting the system stability. Combination with cluster algorithm, similarity, and maximum clique makes it possible to quantify the clique phenomena and monitor them subsequently.

### 2.3 Game process

There are two ways to promote the cooperation between CN and IoT nodes: reward and punishment. It is obvious that cooperation cannot be achieved just with punishment and no reward. However, monitoring is required for selfish nodes, which is energy-consuming. Thus, the question arises whether CN needs to do monitoring or not.

Firstly, we focus on the behavior of cognitive nodes and IoT nodes. Since, the main objective is cooperation and energy saving, the following payoff matrix (Table 2) is constructed by the analysis of energy loss.

**Table 2:** Payoff matrix of interactions between CN and SN nodes.

CN	No monitoring	Monitoring	
SN		Partial monitoring	Full monitoring
Defection	(*I*_*n*_, −*C*_*f*_)	(*I*_*p*_, −*C*_*f*_ − *C*_*pm*_)	(*I*_*f*_ − *P*, −*C*_*f*_ − *C*_*fm*_)
Cooperation	(*I*_*n*_ − *C*_*fc*_, *I*_*gc*_ − *C*_*f*_)	(*I*_*p*_ − *C*_*fc*_ + *R*, *I*_*gc*_ − *C*_*f*_ − *R* − *C*_*pm*_)	(*I*_*f*_ − *C*_*fc*_ + *R*, *I*_*gc*_ − *C*_*f*_ − *R* − *C*_*fm*_)

*I*_*n*_ signifies the income with no monitoring. Likewise, *I*_*p*_, *I*_*f*_ signify that with partial and full monitoring respectively. *P* signifies that the punishment for IoT nodes. It’s shown in Table 3. *C*_*fc*_ signifies that the consumption of finding the channel. *R* signifies that the reward for the contribution of IoT nodes. *C*_*f*_ means that the consumption of forwarding data for IoT nodes. *C*_*pm*_, *C*_*fm*_ mean that the consumption of partial monitoring and full monitoring, respectively. *I*_*gc*_ means that the income of getting channels.

**Table 3:** Relations between direct trust value and punishments.

Direct trust energy	Punishment
$t_{d_{ij}}\geq 0$	no punishment
$0>t_{d_{ij}}\geq -2$	pause three sensing rounds
$-2>t_{d_{ij}}\geq -4$	pause three sensing rounds de-allocate 50% of the reward to the selfish node
$-4>t_{d_{ij}}\geq -6$	de-allocate all reward to this selfish node
$-6\geq t_{d_{ij}}$	de-allocate all reward mark the node as an non-cooperative node and reject to connect with it.

For each unit contribution, there is no difference between the defection portion of incomplete cooperation and defection itself. Similarly, they are the same between the cooperation portion of incomplete cooperation and cooperation itself. On this basis, in Table 2, we define “Defection” as complete non-cooperation, that is to say, those IoT nodes will make no sensing. Likewise, ‘Cooperation’ is defined as complete cooperation.

**Fig 4:**
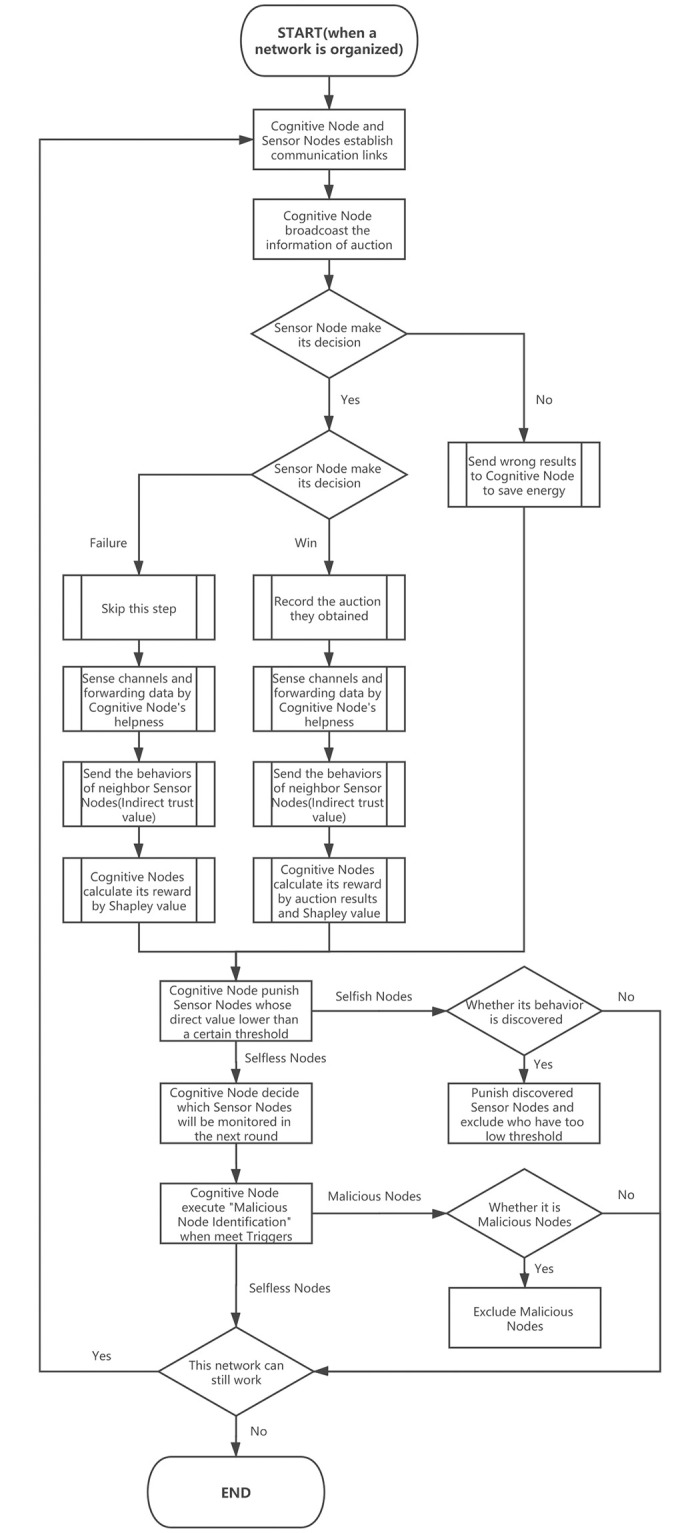
Full process of system model.

In addition, Partial monitoring refers to considering indirect trust value and Full monitoring refers to considering both direct trust value and indirect trust value. (Shown in section 2.5.1), Fig 4.

(1) No monitoring (no reward and no punishment):
  To analyse this topic concisely, we can simplify Tables 2 to 4.
  Given the benefits of selfish behaviors, when CN does not monitor IoT nodes, the dominant strategy of IoT nodes will be **“Defection”**, and **(no monitor, defection)** is the nash equilibrium. This is harmful for the cognitive node and contrary to the rule of global benefit maximization. So we need to take precautionary measures to change this situation. The next issue is which strategy to choose, only motivation, only punishment, or both of them.
(2) With the only reward (no punishment):
  In this item, simplifying Tables 2 to 5 first.
  From the perspective of energy consumption, CN should not use more energy to reward than the required energy used to sense i.e., cognitive node needs to gain positive earnings. That is, *R* + *C*_*f*_ < *I*_*gc*_ and *C*_*fc*_ > *R*.
  Although the earnings from cooperation increase, the dominant strategy is still ‘Defection’. So motivation alone is not enough.
  But, it doesn’t mean that stimulation is utterly useless. First, SNs have some data that they want to send out, which require more energy for transmission in an unlicensed channel. The real values of *R* are not the same for all SNs. Consumption is only a perspective, just more objective, and representative. Besides, this is a repeated game, in the real world, according to experiments in behavioural game theory, we can know that users tend to show their side of goodness when they assume that others are good-intended. Nodes will be inclined to choose cooperation overall.
  Simulation is effective but not enough. Meanwhile, we should consider punishment. Because through this way, we can let the nash equilibrium closer to cooperation.
  The above discussions provide insights in identifying selfish SNs by monitoring.
(3) With both reward and punishment:
  In summary, using both punishment and motivation is more appropriate, like Table 2.
  In this scenario, as the frequency of monitoring increases, the income of choosing to be selfish decreases. When the frequency reaches a number, the income of being selfish or selfless will be the same. Thus, being selfless is a dominant strategy, now the question is how often should cognitive nodes monitor.

**Table 4:** Simplified payoff matrix for ‘No monitoring’ strategy.

CN	No monitoring
SN	
Defection	(*I*_*n*_, −*C*_*f*_)
Cooperation	(*I*_*n*_ − *C*_*fc*_, *I*_*gc*_ − *C*_*f*_)

**Table 5:** Simplified payoff matrix for ‘No punishment’ monitoring strategy.

CN	No monitoring	Partial monitoring
SN		
Defection	(*I*_*n*_, −*C*_*f*_)	(*I*_*p*_, −*C*_*f*_ − *C*_*pm*_)
Cooperation	(*I*_*n*_ − *C*_*fc*_, *I*_*gc*_ − *C*_*f*_)	(*I*_*p*_ − *C*_*fc*_ + *R*, *I*_*gc*_ − *C*_*f*_ − *R* − *C*_*pm*_)

We assume that there exists IoT nodes and a single cognitive node(CN) in the cognitive IoT network which is allowed to access the PU licensed channels with mutual cooperation. The licensed channel is separated into M sub-bands, unlicensed users (CN and SN) can access the vacant M sub-bands in the absence of the licensed user. For the cognitive node to access the licensed channel it needs to sense all the M bands in order to make the access decision. Due to limited battery power, IoT nodes and CNs try to conserve their energy. In the proposed model, the CN distributes sensing tasks to IoT nodes surrounding it and provides them with access to the licensed channel. In this network, we assume that number of IoT nodes is K which are divided into two groups. cooperators (Cg) help in sensing the licensed channel and defectors(Dg) do not help in sensing the licensed channel. The probability of false alarm PF is equal to all IoT nodes.

After one round, the cognitive node will count the contribution of every IoT node. Later, the cognitive node will use shapely values to calculate the assistance’s importance. In the next round, the cognitive node allocates redundant transmission time based on individual contribution. The node whose credit degree is lower than the threshold will be refused to cooperate.

#### 2.3.1 The frequency of monitoring

Monitoring behaviour of IoT nodes is energy-consuming. So the question is how often should we monitor?

Table 2 illustrates that partial monitoring can bring out some benefits, and based on Algorithm 4, we know the energy consumption of indirect trust value is extremely little. With indirect trust value, it can deter selfish behaviors and it is effective. When there is no mechanism to fill up the gap between twice Full monitoring then the frequency of Full monitoring would increase or taking other measures to reduce the number of selfish behaviors. In other words, it reduces the benefit of defection. But these two ways are more energy-intensive than Partial monitor. Therefore, Partial monitoring will run all the time and we will calculate the frequency of Full monitoring in order to save the node energy.

When the expectation of earnings from selfish and selfless behaviors are equal then IoT nodes will have no inclination to be selfish. This is because selfish behaviors will bring no extra earnings compared with selfless behaviors and there is a risk of being regarded as selfish nodes. When a node’s direct trust value (Shown in section 2.5.1) is lower than the threshold then it will be regarded as a selfish node and the cognitive node will refuse to cooperate with it. In this case, defection has become a weakly-dominated strategy.

Further, we decide to calculate frequency by considering no monitoring and full monitoring. Indirect threats are not always effective in the real world by the reason of incompletely rational. In order to achieve our goal mentioned before, thinking about the worst situation is proper. In this sense, the natural corollary is that we can use *I*_*n*_ to replace *I*_*p*_ and *I*_*f*_, and now it’s the income of no sense.

As stated previously, it’s feasible to make Partial monitoring in every time slot. Thus, when we consider the frequency of monitoring, it should be replaced with Full monitoring with the following payoff matrix Table 6 consideration.

**Table 6:** Final payoff matrix with only no monitoring and full monitoring strategies.

CN	No monitoring	Full monitoring
SN		
Defection	(*I*_*n*_, −*C*_*f*_)	(*I*_*n*_ − *P*, −*C*_*f*_ − *C*_*fm*_)
Cooperation	(*I*_*n*_ − *C*_*fc*_, *I*_*gc*_ − *C*_*f*_)	(*I*_*n*_ − *C*_*fc*_ + *R*, *I*_*gc*_ − *C*_*f*_ − *R* − *C*_*fm*_)

In order to make nodes selfless, we should make it impossible to profit by choosing to be selfish. When the income that was obtained by the node to be selfish or selfless are equal then the IoT nodes have no incentive to be selfish. In this sense, their expectations are equal.

Follow the above train of thought, we suppose that the possibility of Full monitoring is *P*, then the possibility of No monitoring is 1 − *P*.

$$E_{defection}=P_{pro}\cdot \left(I_{n}-P\right)+\left(1-P_{pro}\right)\cdot I_{n}$$

$$E_{cooperation}=P_{pro}\cdot \left(I_{n}-C_{fc}+R\right)+\left(1-P_{pro}\right)\cdot \left(I_{n}-C_{fc}\right)$$

$$\begin{array}{c} E_{selfish}=E_{selfless} \end{array}$$ (1)

$$P=\frac{C_{fc}}{R+P}$$

On the basis of previous discussion, we also know:

$$C_{fc}<R+P$$

⇒P<1

Therefore, Eq (1) always has a solution, and only one solution.

The optimal frequency theoretically is $\frac{C_{fc}}{R+P}$.

According to Table 3, *P* is regarded as 3*C*_*fc*_, and *R* will be calculated based on the contribution of last round and extra reward (Section 2.5.2). Moreover, an extra reward can be preset or changed dynamically depending on the situation. These assumptions correspond with real-world scenarios.

### 2.4 Assign sensing tasks

In a IoT network with selfish nodes, when the sensing task gets allocated to the nodes without considering their nature then the tasks assigned for selfish nodes will not be accomplished. Thus, tasks need to be assigned to nodes considering their nature where the auction mechanism meets this requirement.

#### 2.4.1 Behaviors and results of CN and IoT nodes

In the auction mechanism, CN broadcasts the information of assigning tasks and IoT nodes to choose and accept tasks according to their own situation i.e., nature. Generally, selfless SNs tend to accept more tasks.

#### 2.4.2 Auction model for assigning sense tasks

A set of tasks to be assigned is represented as *A* = *A*_1_, *A*_2_……*A*_*m*_ with *m* = 1, 2…… The task which is an auction item is actually reward converted from a basic resource necessity for running tasks. IoT nodes will bid on the resource of running tasks, generally the sensing time, which also means the minimum contribution required of bidders(IoT nodes) in one round. And *B* = *B*_1_, *B*_2_……*B*_*n*_ with *n* = 1, 2….. denotes the bids of IoT nodes. At the same time, IoT nodes have different private values for the same auction item. Due to divergent channel quality, various demand of forwarding, its own residual energy and something else, there is different degrees of importance attached by IoT nodes. *V* = *V*_1_, *V*_2_……*V*_*n*_ with *n* = 1, 2….. is used to express this diversity. Hence, the bids mainly depend on their own private values.

**Fig 5:**
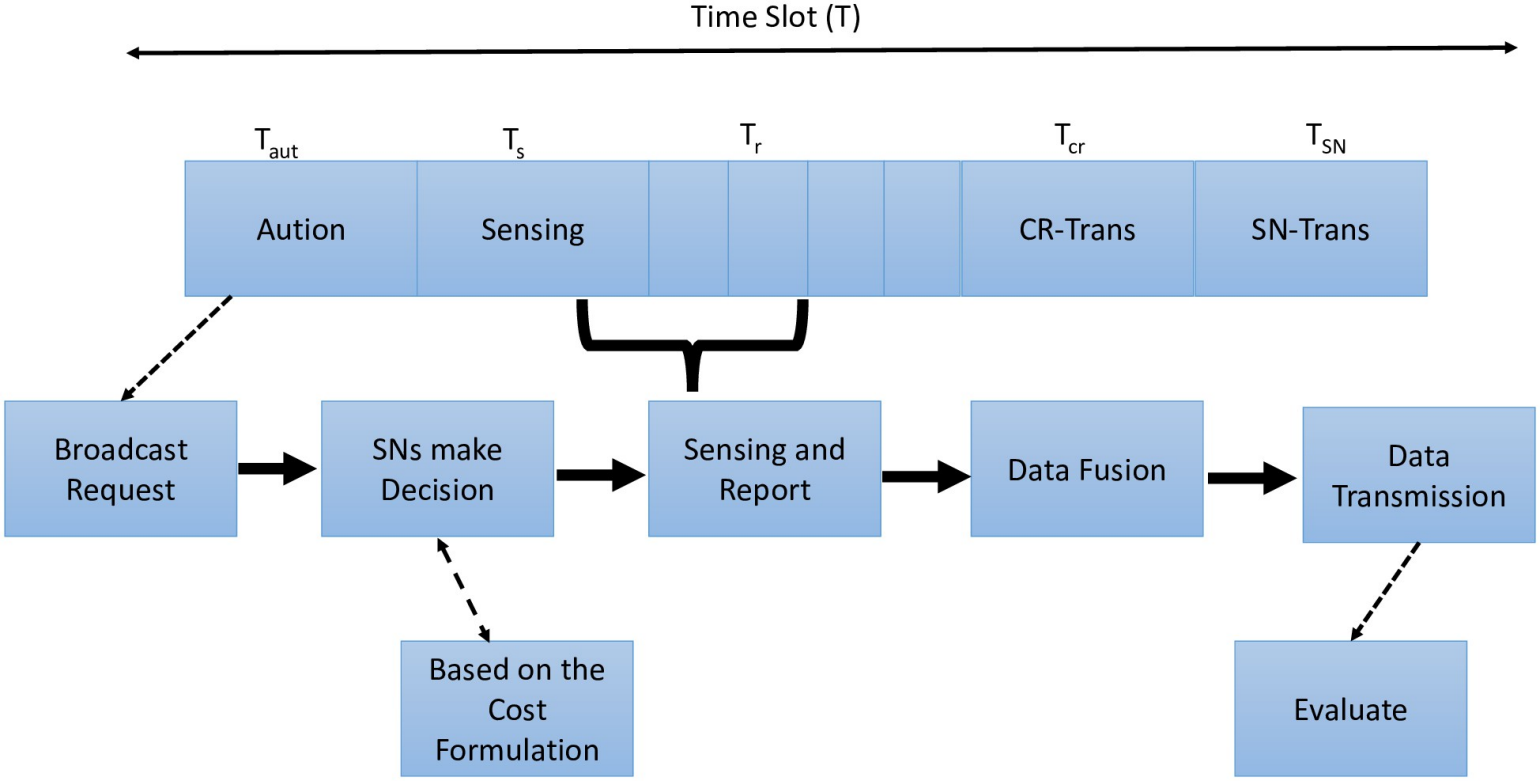
Cognitive-WSN network with selfish and malicious nodes.

Firstly, we consider using an auction game to assign the task of sensing channel. Here, the bid represents the minimum contribution standard of a node. See Table 7 for the details. The Frame format of cognitive nodes auction can be seen in Fig 5. We propose a co-operation promotion mechanism, where each frame is divided into fixed time intervals. At time *T*_*auc*_, cognitive node (CN) conducts auction in multiple direction. The IoT nodes that receive the auction request bid using cost formulation, i.e, based on the energy level, wastage of energy in unlicensed transmission due to high interference. At the second part of the Time frame *T*_*s*_, the cooperating IoT nodes perform PU spectrum sensing. Next, during time *T*_*r*_ the cooperating IoT nodes send data to CN, where CN fuses the data received from the IoT nodes and makes a decision on the channel. When the channel is available, CN transmits its own data during time *T*_*cr*_. While IoT node data is in time *T*_*SN*_, which is the reward for the cooperation.

**Table 7:** Terminology interpretation.

auctioneer	cognitive node
bidders	sense nodes
auction item	cognitive node’s helpness
time	before every unit time
bid	up to tuition

Both CN and IoT nodes desire to reduce energy consumption. Comparing with the sensing channel by CN itself, it will not consume more required energy to reward in the auction stage. In this sense, CN will be motivated to hold an auction only when it can save its energy after this stage. Further, CN will not accept the bids which are lower than the required energy that CN senses channel by its own self. This is the lower bound of bids for every SN. Similarly, IoT nodes also hope that they can cost less energy than forwarding in unlicensed channels. We assume that the bids of IoT nodes obey Normal Distribution subjected to [*E*_*l*_, *E*_*ul*_] whereas *E*_*l*_, *E*_*ul*_ represent the energy cost in licensed or unlicensed channel (Section 2.4.3).

For the discussion of Section 2.4.4-*Lemma*2, we decide to add reserve price in the early stage and call it off in the later.

#### 2.4.3 Energy consumption analysis

The consumption of one transmission is as follows:

$$\begin{array}{c} E^{TX}\left(L,d\right)=\{\begin{array}{c} E_{tx}\cdot L+\epsilon_{fs}\cdot L\cdot d^{2},\;d\leq d_{0}\\ E_{tx}\cdot L+\epsilon_{mp}\cdot L\cdot d^{4},\;d>d_{0} \end{array} \end{array}$$ (2)

$$d_{0}=\sqrt{\frac{\epsilon_{fs}}{\epsilon_{mp}}}$$

*d* is distance. *L* is the number of bits. *E*_*tx*_ is the transmit energy consumed per bit. *ϵ*_*fs*_ refers to free-space power loss and *ϵ*_*mp*_ refers to multi-path fading channel.

In our case, *E*_*tx*_, *ϵ*_*fs*_, *ϵ*_*mp*_, and *d* are constants.

Given the possibility of transmitting unsuccessfully, the total energy consumption for one transmission is equal to “the energy consumption of one transmission ⋅ (re-transmitting number + 1)”.

Here, *E*_*total*_ = *E*_*TX*_ ⋅ (*m* + 1). *m* is different for forwarding package in licensed channel and unlicensed channel.

The probability of re-transmitting in the licensed channel is 30%. Therefore, the number of re-transmissions *m*_*l*_ is looked upon as $\sum_{i=1}^{\infty }0.3^{i}=0.3/0.7=0.43$(also equal to $1-\frac{1}{1-0.3})$.

The probability of re-transmitting in the licensed channel is given by the following formula ([25]):

$$P_{o}=\frac{E_{\pi }\left[1_{\left\{S=0\right\}}\right]}{E_{\pi }\left[W\right]}$$

$$m_{l}=\sum_{i=1}^{\infty }P_{o}^{i}=0.3/0.7=0.43\left(also\;equal\;to\;1-\frac{1}{1-P_{o}}\right)$$

*m*_*l*_ represents the number of re-transmissions. *P*_*o*_ shows the probability of re-transmission timeout. Another aspect of forwarding is transmitting in the unlicensed channel. The transmissions formula for unlicensed channel is as follow ([26]):

$$\begin{array}{c} \begin{array}{c} m_{ul}=\underset{m\in N}{max}\;log\left(-\frac{1}{k\lambda }log\left(1-\epsilon^{\frac{1}{m+1}}\right)\right)+\\ \alpha {\left(-\frac{1}{k\lambda }\right)}^{\frac{\alpha }{2}}\;\frac{{\left(log\left(1-\epsilon^{\frac{1}{m+1}}\right)\right)}^{\frac{\alpha }{2}-1}}{2-\frac{2}{k\lambda }(log{\left(1-\epsilon^{\frac{1}{m+1}}\right)}^{\frac{\alpha }{2}}} \end{array} \end{array}$$ (3)

$$k=\pi r_{0}^{2}\Gamma \left(1-\frac{2}{\alpha }\right)\Gamma \left(1+\frac{2}{\alpha }\right)$$

*m* is the attempts. *α* is the path loss exponent. λ represents the network density. *ϵ* represents the maximum acceptable error rate. The channel quality of licensed channels should be higher than that of unlicensed channels. Namely, the expected number of re-transmission in an unlicensed channel is supposed to be larger than the licensed channel. Therefore, *m*_*ul*_ > 0.43 always stand up. In summary, the total consumption is

$$\begin{array}{c} E_{total}=\{\begin{array}{c} 1.43\cdot (E_{tx}\cdot L+\epsilon_{mp}\cdot L\cdot d^{4}),\;\;\;\\ when\;using\;licensed\;channels\;\;\\ \left(m_{ul}+1\right)*\left(E_{tx}\cdot L+\epsilon_{mp}\cdot L\cdot d^{4}\right),\;\;\\ when\;using\;unlicensed\;channels \end{array} \end{array}$$ (4)

#### 2.4.4 The lemma of auction model

**Lemma 1**. *All bidders will bid on their ideas without cheating*.

**Lemma 2**. *At first, it is beneficial for the cognitive node to set a reserve price. But if the energy of IoT nodes is low and some IoT nodes may choose to be out of this auction, the cognitive node should call reserve price off*

Proof: The basic settings are shown in Table 7. The basic assumptions are shown in the following.

(1) Private price: All IoT nodes have its private value, this value isn’t affected by other nodes value and it is unknown to other nodes.
(2) Independence of variables:Point distribution function of *v*_1_, *v*_2_…*v*_*k*_ is:
  $$F\left(v_{1},v_{2}\ldots v_{k}\right)=F\left(v_{1}\right)\times F\left(v_{2}\right)\ldots .\times F\left(v_{k}\right)$$
(3) For ∀*i*, *j*, *F*(*v*_*i*_) = *F*(*v*_*j*_)
(4) All IoT nodes are risk-neutral
(5) Variation: SNs decide its bid value without the influence from other tnode’s and the bid is continuous variables, so there is very little chance of tied bids.
(6) Tie Situation: when two or more nodes offer the same bids, the auction item will be randomly assigned to a node.

Moreover, to some extent, IoT nodes are risk-averse. In another way, multi-auctions will make IoT nodes that do not get any auction item anxious. Due to that, a node which is risk-averse will offer a higher bid than that which is risk-neutral. Hence, this is better for the cognitive node. Thus, we can think of risk-averse SNs as risk-neutral with no negative impact on CN.

In this model, the expected income of IoT node *i* is

$$\begin{array}{c} E_{I_{i}}=\left(b_{i}-b_{\left(2\right)}\left(i\right)\right)\cdot Pr\left(\forall j,b_{i}>b_{j},i\neq j\right) \end{array}$$ (5)

*b*_(2)_ means the second highest bid. And as mentioned before, for this model, the best strategy is *f*_*i*_(*v*_*i*_) = *b*_*i*_ = *v*_*i*_. So Eq 5

$$E_{I_{i}}=\left(v_{i}-b_{\left(2\right)}\left(i\right)\right)\cdot Pr\left(\forall j,b_{i}>b_{j},i\neq j\right)$$

Given if a IoT nodes’ private price is *a*, it can’t offer a price lower than *a*, so let *f*_*i*_(*a*) = *a*.

For briefness, assuming *b*_*i*_ ∈ [0, 1]. This is because that any interval can be generalized by (In our paper, we use $\frac{1}{b-a}x-\frac{a}{b-a}$).

Support that the best reserve price has been worked out. Its result is the solution of

$$\begin{array}{c} R-\frac{1-F(R)}{f(R)}=v_{0} \end{array}$$ (6)

*R* represents the reserve price. *v*_0_ signifies the private value of the cognitive node.

So the next question is: What’s the value of *v*_0_?

Sensing nodes need to forward their data with the cognitive node’s help while the cognitive node is looking forward to saving energy through the perception of IoT nodes. Because of the cognitive node’s monopoly position in the aspect of forwarding data, the cost of channel sensing must be more than or equal to the energy for the data that need forward by the cognitive node. Meanwhile, it is reasonable to assume that all IoT nodes always have data to forward, so such a transaction will always exist.

The following conclusions can be drawn from the above: for IoT nodes, it bids should be more than and equal to the energy used to forward(their value on the interval [*a*, *b*]). For the cognitive node, its reserve is equal to the cost used to help IoT nodes. In other words, it’s *a*. At the same time, we assume *a* is 0. It is possible to state that the conclusion is:

$$v_{0}=0$$

F(R)=R,f(R)=1,R∈[0,1]

$$\Rightarrow R=\frac{1}{2}$$

$$\Rightarrow R=\frac{a+b}{2}=\frac{\left(m_{ul}+2.6\right)*\left(E_{tx}\cdot L+\epsilon_{mp}\cdot L\cdot d^{4}\right)}{2}$$

This moment, the expected payment of IoT node is

$$f_{i}\left(v_{i}\right)=\frac{k-1}{k}\cdot v_{i}+\frac{{\frac{1}{2}}^{k}}{kv_{i}^{\left(k-1\right)}}$$

The expected income of cognitive node is

$$E_{I_{CN}}\left(k\right)=\frac{k-1}{k+1}+\frac{1}{\left(k+1\right)\cdot 2^{k}}$$

**Lemma 3**. *Because participating in the auction will consume energy, some IoT nodes will not choose to take part in the auction if they think the reserve price is high and they can’t win. So when there are n IoT nodes participate in this auction, then the expectation is*

$$\begin{array}{c} E_{I_{CN}}\left(n\right)=\frac{n-1}{n+1}+\frac{1}{\left(n+1\right)\cdot 2^{n}} \end{array}$$ (7)

In the light of the formula (7), we can know that the more nodes join, the more income the cognitive node can gain. But cancelling the reserve price will lose some benefits. So the question is changed to, “Is the lost income by cancelling the reserve price more than benefit from more nodes participating”. Consider an extreme case, is it still worth if only one more node participates? $E_{I_{CN}}\left(n+1\right),E_{I_{CN}}\left(n+2\right),E_{I_{CN}}\left(n+3\right)\ldots .$ means the expectation which don’t involve reserve price.

$$\Leftrightarrow E_{I_{CN}}\left(n+1\right)>E_{I_{CN}}\left(n\right)$$

$$\Leftrightarrow \frac{n}{n+2}>\frac{n-1}{n+1}+\frac{1}{\left(n+1\right)\cdot 2^{n}}$$

$$\begin{array}{c} \Leftrightarrow 2^{k+1}>k+2 \end{array}$$ (8)

For *k* ≥ 1, formula (5) is always true. And $\frac{n-1}{n+1}$ is monotone increasing, so as long as eliminating the reserve price makes the auction more crowded its justifiable.

### 2.5 Cooperation mechanism

**Fig 6:**
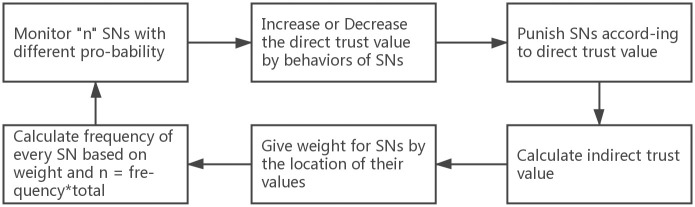
Identify selfish nodes.

To improve cooperation and robustness, the core issue is with selfish nodes. First, CN should choose which strategies need to be selected. Thus, we proposed mechanisms for strategies selected separately can be seen in Fig 6. Later, the case that majority of nodes are selfish is considered final.

#### 2.5.1 Trust value

Before diving into the frequency of monitoring, quantitative analysis for cooperation need to be solved, That is, the rewards and punishments process and rules. We can calculate and analyse the payoffs in various cases where monitor mechanisms and risks are clearly stated.

In this section, we describe the proposed method, which deals with the challenge that, most of the IoT nodes only provide information about a few channels.

(1) Direct trust value:
  In every round, the cognitive node decides to monitor at the frequency which has been calculated at the end of the last round by 2.3.1. In round 1, the cognitive node monitors all IoT nodes.
  When the cognitive node finds nodes as selfish behavior, then the IoT node’s direct trust value will be reduced by 1. Otherwise, the value will be increased by 1.
  $$t_{d_{new}}=\{\begin{array}{cc} t_{d_{last}}+1 & \;\;Cooperate\\ t_{d_{last}}-2 & \;\;Defect \end{array}$$
  We program multi-threshold in order to make the system more fair and rational. (have reduced similarity)
(2) Indirect trust value:
  Sense nodes have their interactions, so they have their own comments on other nodes. To make our model fair, we should consider these comments. In our paper, it is called ‘indirect trust value’
  $$N_{in_{new}}=\{\begin{array}{cc} N_{in_{last}}+1 & \;\;Cooperate\\ N_{in_{last}}\; & \;\;Defect \end{array}$$
  $$Pro_{ij}=\frac{N_{c}}{N_{c}+N_{d}}$$
  $$\begin{array}{c} t_{in_{ij}}=N_{ij}*{\left(Pro_{ij}\right)}^{2} \end{array}$$ (9)
  $$t_{in_{ii}}=0$$
  We assume that the amount of successful cooperation is *N*_*c*_. On the contrary, the amount of selfish behaviors is *N*_*d*_. *Pro*_*ij*_ indicates the probability of being trusted.
  In this scenario, as selfish behavior happens, the *Pro* reduces. It means, the $t_{in_{ij}}$ also reduces.
  In addition, using Eq (9) is propitious to prompt interactions among IoT nodes because the number of interactions is a key parameter in the calculation of indirect trust value.
  $$T_{in_{ij}}=\frac{t_{in_{ij}}}{\sum_{j=1}^{N}t_{in_{ij}}}$$
  $${\text{t}}_{\text{in}}=\left(t_{in_{1}},\;t_{in_{2}}\ldots \ldots \;t_{in_{n}}\right)$$
  And assuming
  $$t_{in_{0}}=\left(1/n,\;1/n\ldots \ldots \;1/n\right)$$
  $${\text{t}}_{\text{all}}=[\begin{array}{cccc} T_{in_{11}} & \;T_{in_{12}} & \ldots \ldots & \;T_{in_{1n}}\\ T_{in_{21}} & \;T_{in_{22}} & \ldots \ldots & \;T_{in_{2n}}\\ \;\ldots \ldots & \;\ldots \ldots & \ldots \ldots & \;\ldots \ldots \\ \;\ldots \ldots & \;\ldots \ldots & \ldots \ldots & \;\ldots \ldots \\ T_{in_{n1}} & \;\ldots \ldots & \ldots \ldots & \;T_{in_{nn}} \end{array}]$$
  This matrix is convergent. (With the continuous implementation of our system, the behaviors will tend to be selfish. It will cause smaller standard deviation and accelerate convergence rate.) Assuming the convergence matrix is following:
  $${{\text{t}}_{\text{all}}}^{\prime }=[\begin{array}{cccc} {T_{in_{11}}}^{\prime } & \;{T_{in_{12}}}^{\prime } & \ldots \ldots & \;{T_{in_{1n}}}^{\prime }\\ {T_{in_{21}}}^{\prime } & \;{T_{in_{22}}}^{\prime } & \ldots \ldots & \;{T_{in_{2n}}}^{\prime }\\ \;\ldots \ldots & \;\ldots \ldots & \ldots \ldots & \;\ldots \ldots \\ \;\ldots \ldots & \;\ldots \ldots & \ldots \ldots & \;\ldots \ldots \\ {T_{in_{n1}}}^{\prime } & \;\ldots \ldots & \ldots \ldots & \;{T_{in_{nn}}}^{\prime } \end{array}]$$
  $$\begin{array}{c} {\text{t}}_{\text{in}}=t_{in_{0}}*{{\text{t}}_{\text{all}}}^{\prime } \end{array}$$ (10)
  It’s a sparse matrix. So we plan to smooth it first. Therefore, we change (10) into
  $${\text{t}}_{\text{in}}=t_{in_{0}}*{\left(\alpha *I+\left(1-\alpha \right)*{\text{t}}_{\text{all}}\right)}^{n}$$
  *I* is the identity matrix.
  The percent of *nodei* points that the location of $t_{{\text{in}}_{\text{i}}}$ in all direct trust values when they are from smallest to largest. Because a node with higher $t_{in_{i}}$ is less possible to choose selfish behaviors, the lower weight (Table 8) is given to them.
  $$fre_{new}=w\left(t_{d_{ij}}\right)*\;fre$$

**Table 8:** Standard for assigning weights.

	location	weight
*w*_*ij*_ =	0% ∼ 25%	1.75
	25% ∼ 50%	1.25
	50% ∼ 75%	0.75
	75% ∼ 100%	0.25

#### 2.5.2 Reward(Shapely)

Apart from the sensing task assigned in the auction slot, SNs are capable to do additional sensing provided they get a satisfactory reward. This is the result, CN wants to achieve(SNs can be motivated to sense channel by reward). Besides, the conclusion of Section 2.3 has universality. Given that the punishment mechanism is applied throughout all time slots, the reward for extra contribution is necessary.

We suppose that the number of participators is *I* = {1, 2, 3……*n*}, *s* is a subset of *I*, *l*_(_*s*) is the reward for this subset, *φ*_*i*_(*l*) is the reward of i, *φ*(*l*) = (*φ*_1_(*l*), *φ*_2_(*l*)……*φ*_*n*_(*l*)) is the total lucre, |*s*| means the cardinal number of *s*.

It’s obvious that *l*(∅) = 0 and *l*(*s*_1_ ⋃ *s*_2_) ≥ *l*(*s*_1_) + *l*(*s*_2_). When a model can meet the following four axioms, it must have a unique solution of Shapely value.

*Axiom*1: Assuming *π* is a permutation of *I*, *s* = {1, 2……*i*}, *πs* = {*π*1, *π*2……*πi*}, we can draw a conclusion that *φ*_*πs*_(*πl*) = *φ*_*s*_(*l*) must be accurate. Say in other words, the lucre of a participator isn’t related to its rank.

*Axiom*2:

$$\sum_{i\subset I}\varphi_{i}\left(l\right)=l\left(I\right)$$

*Axiom*3: for *S*_*i*_ = {*s*_*j*_|*i* ∈ *s*_*j*_, *s*_*j*_ ⊂ *I*}. If ∀*s*_*j*_, *l*(*s*_*j*_\*i*) = *l*(*s*_*j*_) ⇒ *φ*_*i*_(*l*) = 0

*Axiom*4: *φ*(*l*_1_) + *φ*(*l*_2_) = *φ*_(_*l*_1_ + *l*_2_) The reward will be figured out by *formula*(11).

$$\begin{array}{c} \varphi_{i}\left(l\right)=\sum_{l(s)\subset I}\frac{(n-|s|)!\cdot (|s|-1)!}{n!}\cdot \left[l\left(s\right)-l_{-i}\left(s\right)\right] \end{array}$$ (11)

After each round, the CN uses the ‘Shapely value’ to assign a reward for each node based on its contribution. Subsequently, within the next round, each SN can get access to forwarding data in the licensed channel and limit the total data volume to no more than overall the reward obtains from auctions and shapely value phase.

The final value of the contribution is calculated by *x*^1.5^. *x* is sensing time that a certain SN devotes for CN(The setting of ‘1.5’ makes the marginal utility of making one more unit sensing increases).

Although this model with such an assumption can be replaced in another way, it’s still worth using it to provide a framework and cost less energy with simple assumptions.

Additionally, to balance the consumption of the whole network, it is necessary to consider the remaining energy of every node and give them weight based on it. Because our purpose is balance, scilicet, lets each node contribute the same amount of energy as much as possible. On the basis of Section 2.5.2, We set

$$w\left(i\right)=\frac{E_{total}}{E_{remain)}\left(i\right)}$$

$$\Rightarrow l{\left(i\right)}^{\prime }=l\left(i\right)w\left(i\right)$$

$$\Rightarrow △l\left(i\right)=l{\left(i\right)}^{\prime }-\frac{1}{n}$$

$$\Rightarrow △\varphi_{\left(1\right)}\left(l\right)=\varphi_{\left(1\right)}\left(l\right)*l\left(i\right)*\alpha \;\left(0<\alpha <1\right)$$

$$\varphi_{\left(1\right)}{\left(l\right)}^{\prime }=\varphi_{\left(1\right)}\left(l\right)+△\varphi_{\left(1\right)}\left(l\right)$$

#### 2.5.3 Punish for selfish IoT nodes

The specific measures are shown in Table 3. And MP(Monitoring and punishing process) process is presented as follows:

(1) Sensing nodes do bidding in the auction and sensing for CN and send results.
(2) Bad IoT nodes send wrong results to CN(other nodes can observe this information) to save energy.
(3) When neighbors observe this information they store it in their database as correct information or wrong information.
(4) Neighbor nodes send their data and information about its neighbors to CN.
(5) CN analyzes this information over a period of time to find selfish behaviors and punish those SNs.

In our paper, there are two trust value models to evaluate the character of IoT nodes and decide how to deal with them. Direct trust value is gained by the cognitive node and used to give weight to the frequency we identified before. Indirect trust value is from the interactions of IoT nodes and the role in analyzing the character of IoT nodes.

At the same time, if the direct trust value is lower than the threshold that we predetermined then the cognitive node will refuse to cooperate.

### 2.6 Identify malicious nodes

The reliability of indirect trust value will decrease with the increase in the number of selfish SNs and these SNs are more likely to form cliques and cheat CN together. Meanwhile, the absence of a mechanism to regulate such cases will reduce the robustness of the system.

In this paper, we define “**malicious node**” as a IoT node that provides false indirect trust value to cognitive, that is, giving members of its clique high values but low values to others deliberately.

In the context of such a scenario, selfish nodes will be given low indirect trust values by other IoT nodes and the frequency of monitoring from cognitive will increase. But if there is no strategy to identify fake values and punish providers, selfish nodes are tilting toward to form a clique and be malicious nodes.

Accordingly, it’s indispensable to build a model to prevent IoT nodes from being malicious nodes. To this end, we design the following method:

(1) Clustering indirect trust values:
  First, *K*–*meansClusteringalgorithm* will be used to classify them into two groups: *highvaluegroup* and *lowvaluegroup*. Then, the initial point is set between 0.05 and 0.95.
  In contrast to other clustering algorithms, it costs lower energy and it’s faster by reason of simplicity. It’s not wealthy to achieve a better clustering with much more energy consumption. The main weakness with this algorithm is following:
  1. In general, people don’t know clearly how many initial points we should cluster.
  2. It’s difficult to learn which point should be confirmed at the beginning.
  3. It’s an input-sensitive algorithm for outliers, noises, isolated points, and so on.
  In the context of our paper, we should cluster these values into two groups and we know the ballpark values of initial points. Besides, the outliers things are very rare and have very small negative effects on the system except leaving a few malicious nodes out or adding selfless nodes wrongly, but they do not matter. From what has been discussed above, we can reasonably arrive at the conclusion that *K*–*meansClusteringalgorithm* is a proper choice.
(2) Quantify and analyze:
  In the beginning, we quantify the relationship between IoT nodes and these two groups by the following function.
  $$g\left(t_{in_{ij}}\right)=\{\begin{array}{cc} 1 & \;\;t_{ij}\in high\;value\\ 0 & \;\;t_{ij}\in low\;value \end{array}$$
  $$\Rightarrow {\text{nt}}_{\text{i}}=\left\{g\left(t_{i1}\right),g\left(t_{i2}\right),\ldots \ldots ,g\left(t_{in}\right)\right\}$$
  Due to the aforementioned feature of malicious nodes, the IoT nodes in the same clique will have the same indirect trust value table. In this context, we can analyze through calculating the similarity of the value table among these nodes. ‘Cosine Similarity’ is an attempt to account for the similarity between two vectors.
  $$Similarity_{i,j}=\frac{{\text{nt}}_{\text{i}}*{\text{nt}}_{\text{j}}}{\left\|{\text{nt}}_{\text{i}}\|\|{\text{nt}}_{\text{j}}\right\|}=$$
  $$\frac{\sum_{k=1}^{N}g\left(t_{ik}\right)*g\left(t_{jk}\right)}{\sqrt{\sum_{k=1}^{N}g{\left(t_{ik}\right)}^{2}}\;\sqrt{\sum_{k=1}^{N}g{\left(t_{jk}\right)}^{2}}}$$
(3) Clique partition:
  Thirdly, regarding all nodes as points in a graph and creating connections lines between two points if their similarity is more than 90%. Thus, it’s easy to find the cliques in this graph, and the cognitive node will choose a node in every clique, with members nodes larger than 5 randomly, to judge whether it’s a malicious node. If so, the cognitive node will monitor all nodes in this clique. Otherwise, the cognitive node will stop monitoring.
  When the number of members in a clique is smaller than 5, we need not care about it. It is possible that there are a few selfless neighbor nodes and they communicate with similar SNs. As a result, those neighbor SNs have similar behaviors, trust values, and so forth although they are willing to cooperate with CN.

## 3 Algorithm

### 3.1 Auction

**Algorithm 1** The Second Price Saled Auction

**Require**: the bid of IoT nodes

**Ensure**: the price (forward data amount)

 1: **while** the beginning of every round **do**

 2:  Cognitive node calculates its budget for the arriving task which will be treated as auction items and reserve nodes.

 3:  The auctioneer invites the public bidding, broadcasts starting price for auction items.

 4:  Sense nodes compare it with remain energy to decide whether to participate or not and calculate the cost of unlicensed channel based on our proposed cost formulation.

 5:  The seller sets the bid value.

 6:  **if** If at least one bid is higher than reserve price **then**

 7:   The winner would be the seller with the highest bid value and pay with the second-lowest bid.

 8:   The auctioneer allocates the task to the winner.

 9:  **else**

 10:   CN will give up this cooperation.

 11:  **end if**

 12:  Then the node winners fuse data followed by CN and SN data transmission.

 13:  CN rewards these SNs with transmission time.

 14: **end while**

### 3.2 Shapely value

**Algorithm 2** shapely value

**Require**: the contribution of IoT nodes in once round and the remaining energy

**Ensure**: the end of every round

 1: **while** the reward for IoT nodes **do**

 2:  $\varphi_{i}\left(l\right)=\sum_{l(s)\subset I}\frac{(n-|s|)!\cdot (|s|-1)!}{n!}\cdot \left[l\left(s\right)-l_{-i}\left(s\right)\right]$

 3: $w\left(i\right)=\frac{E_{total}}{E_{remain)}\left(i\right)}$

 4: *l*(*i*)′ = *l*(*i*)*w*(*i*)

 5: $△l\left(i\right)=l{\left(i\right)}^{\prime }-\frac{1}{n}$

 6: △*φ*_(1)_(*l*) = *φ*_(1)_(*l*) * *l*(*i*) * *α* (0 < *α* < 1)

 7: *φ*_(1)_(*l*)′ = *φ*_(1)_(*l*) + △*φ*_(1)_(*l*)

 8: **end while**

   **return** *φ*_(1)_(*l*)′

### 3.3 Direct trust

**Algorithm 3** direct trust value

**Require**: behavior of *node*_*ij*_, *fre*

**Ensure**: punishment

 1: i = 1:n

 2: j = 1:n

 3: syms $t_{d_{0}}$, $t_{d_{ij}}$

 4: $t_{d_{0}}\;=\;0$

 5: $t_{d_{ij}}\;=\;t_{d_{0}}$

 6: **for** cognitive nodes decide to do full monitor **do**

 7:  **if** find selfless behvior **then**

 8:   $t_{d_{ij}}=t_{d_{ij}}+1$

 9:  **else**

 10:   $t_{d_{ij}}=t_{d_{ij}}-2$

 11:  **end if**

 12:  update direct trust values table

 13:  **if** $t_{d_{ij}}\geq 0$ **then**

 14:   no punishment

 15:  **else if** $0>t_{d_{ij}}\geq -2$ **then**

 16:   give a timeout for three sensing rounds

 17:  **else if** $-2>t_{d_{ij}}\geq -4$ **then**

 18:   give a timeout for three sensing rounds

 19:   de-allocate 50% of the assigned resources to the misbehaving node

 20:  **else if** $-4>t_{d_{ij}}\geq -6$ **then**

 21:   de-allocate all resources and disconnect this node

 22:  **else** $-6\geq t_{d_{ij}}$

 23:   mark the node as an undesirable node

 24:   de-allocate all resources and disconnect this node

 25:  **end if**

 26: **end for**

 27: **while** condition **do**

 28:  …

 29: **end while**

   **return** *e*

### 3.4 Indirect trust

**Algorithm 4** indirect trust value

**Require**: the interaction among IoT nodes, *α*

**Ensure**: **t**_**in**_ and *w*_*ij*_

 1: i = 1:n

 2: j = 1:n

 3: *α* = 0.05

 4: syms $N_{in_{0}}$, $N_{in_{ij}}$, *Pro*_*ij*_, *t*_*inij*_, $T_{in_{ij}}$, ${\text{t}}_{{\text{in}}_{0}}$, $t_{{\text{in}}_{\text{ij}}}$

 5: $N_{in_{0}}=0$

 6: $t_{in_{ii}}=0$

 7: $t_{in_{0}}=\left(1/n,\;1/n\ldots \ldots \;1/n\right)$

 8: **for** interaction happens **do**

 9:  **if** another node is trusted **then**

 10:   $N_{in_{ij}}=N_{in_{ij}}+1$

 11:  **else**

 12:   $N_{in_{ij}}=N_{in_{ij}}-1$

 13:  **end if**

 14: **end for**

 15: $Pro_{ij}=\frac{N_{c}}{N_{c}+N_{d}}$

 16: $t_{in_{ij}}=N_{ij}*{\left(Pro_{ij}\right)}^{2}$

 17: $T_{in_{ij}}=\frac{t_{in_{ij}}}{\sum_{j=1}^{N}t_{in_{ij}}}$

 18: *sum* = *sum*(*matrix*, 2)

 19: **For** i = 1:n

 20:  **For** j = 1:n

 21:   a(ij) = a(ij) / sum(i)

 22:  **End**

 23: **End**

 24: **t**_**all**_′ = *α* * *I* + (1 − *α*) * **t**_**all**_

 25: n = 2

 26: **while** *matrix*′! = *matrix*^*n*^ **do**

 27:  **t**_**all**_′ = **t**_**all**_′ * (*α* * *I* + (1 − *α*) * **t**_**all**_)

 28: *n* = *n* + 1

 29: **end while**

 30: Sort

   **return t**_**in**_, *w*_*ij*_

### 3.5 Malicious

**Algorithm 5** identify malicious node

**Require**: tables from IoT nodes

**Ensure**: clique

 1: **for** each IoT node ∈ all nodes C **do**

 2:  *s*_*i*_ ← *e*_*i*_ ∈ *E*(random)

 3: **end for**

 4: **for** each *e*_*i*_ ∈ *E* **do**

 5:  *l*(*e*_*i*_) ← *argminDistance*(*e*_*i*_, *c*_*j*_), *j* ∈ 1,2…k

 6: **end for**

 7: changed ← false

 8: iter ← 0

 9: **while** until changed true and iter < Mazlters **do**

 10:  **for** each *s*_*i*_ ∈ S **do**

 11:   UpdateCluster(*c*_*i*_)

 12:  **end for**

 13:  **for** each *e*_*i*_ ∈ E **do**

 14:   minDist ← argminDistance(*e*_*i*_, *c*_*j*_), j ∈ 1,2…k

 15:   **if** m **then**inDist l(*c*_*i*_)

 16:    *l*(*e*_*i*_) ← minDist

 17:   **end if**

 18:   changed ← true

 19:  **end for**

 20:  iter++

 21: **end while**

 22:  give all nodes by 0/1

 23:   calculate *Similarity*_*i*,*j*_ =

$\frac{\sum_{k=1}^{N}g\left(t_{ik}\right)*g\left(t_{jk}\right)}{\sqrt{\sum_{k=1}^{N}g{\left(t_{ik}\right)}^{2}}\;\sqrt{\sum_{k=1}^{N}g{\left(t_{jk}\right)}^{2}}}$

 24: k = 2

 25: **while** k **do** ≤ 5

 26:  group all IoT nodes in pairs

 27:  merge k-cliques into (k+1)-cliques

 28:  remove duplicates

 29:  print all 5-cliques and monitor them

 30: **end while**

   **return** result

## 4 Simulation setup and performance analysis

In this section, we test the PR-CSDF mechanism. We consider a cognitive radio network with one CR (SU) and multiple IoT nodes.

- The packet arrival rate λ is set to 0.1 packet/ms, and the packet is length is 1000 bytes with the deadline of 40 ms. The bandwidth of the channel is assumed to be 1 MHz, the CR uses 2mW of transmit power.
- Next, the frame size of sensing is 100 ms. Each time frame is divided into 10-time slots, where slots are used for sensing.
- In the first slot, an auction is conducted for the distribution of sensing tasks, in the second slot, sensing is performed by IoT nodes, in the third slot, IoT nodes send sensing reports, followed by CR and SN transmissions.
- After the computation, the value of reserved prices is set between 0.2 and 0.82. We initially start with 0.2 and increases with the step size of 0.005.

Simulation Environment: The experiments were conducted in a simulated environment replicating a cognitive radio-assisted IoT network scenario. Parameters such as network size, node distribution, and communication protocols were defined to mimic real-world IoT deployments. Implementation of PR-CSDF: The PR-CSDF approach, comprising the distribution of sensing tasks among IoT nodes and the cooperation enhancement through punishment-reward strategies, was implemented in the simulation environment.

Task Distribution: The cognitive node acted as an auctioneer, broadcasting sensing tasks to neighboring IoT nodes. Rational IoT nodes, acting as bidders, sent bids indicating the amount of sensing time they were willing to offer in exchange for rewards from the cognitive node.

Payoff Analysis: The payoff of both the cognitive node and IoT nodes was analyzed to determine the upper and lower bounds of feasible bids based on energy consumption considerations in licensed and unlicensed channels.

Game Theory Model: A game theory model was developed to capture IoT node behaviors (selfish or selfless) and their corresponding payoffs. This model guided the application of reward and punishment strategies to incentivize cooperation among IoT nodes.

Optimal Monitoring Frequency: An optimal monitoring frequency formula was presented to guide the cognitive node in efficient energy utilization while dynamically adjusting monitoring frequencies in each round.

Cooperation Mechanism: The cooperation process, including reward and punishment mechanisms, was implemented. Indirect trust values, additional sensing rewards, and punishment for selfish IoT nodes were integrated into the mechanism to promote cooperation and deter selfish behaviors.

**Fig 7:**
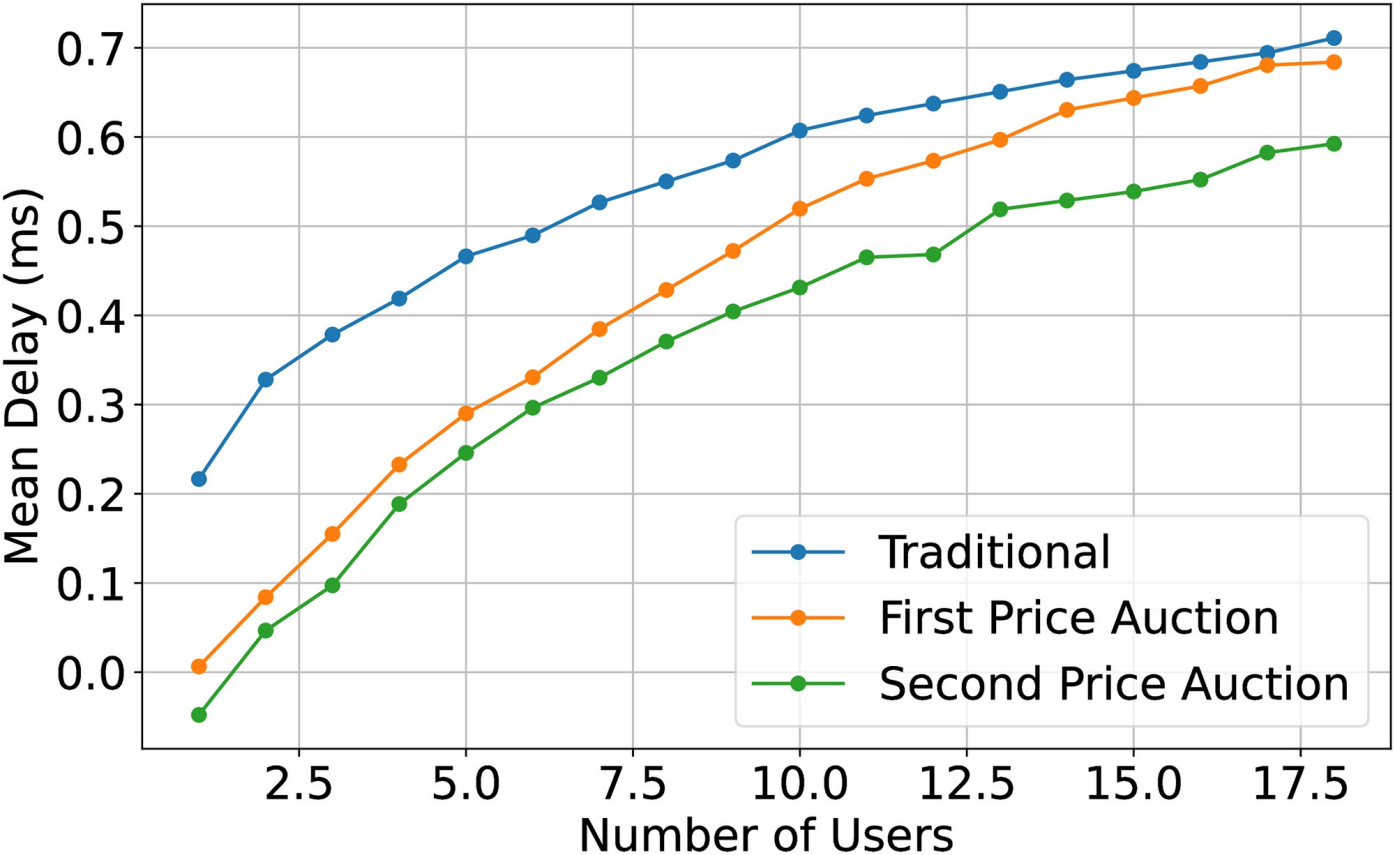
Mean delay of three task distribution and sensing model.

In Fig 7, the rate of packet delay is plotted against the number of IoT nodes for the first-price and second-price auction. Both, the auction schemes perform comparatively well than individual sensing. It is observed that the delay for traditional sensing is higher compared to both the second and first price auctions. This can be justified by the auction mechanism’s ability to efficiently allocate sensing tasks among IoT nodes, leading to a more optimized and coordinated sensing process. The auction mechanism ensures that tasks are assigned based on bids, which can result in a more streamlined and timely execution of sensing activities compared to traditional methods where tasks may not be allocated as efficiently.

**Fig 8:**
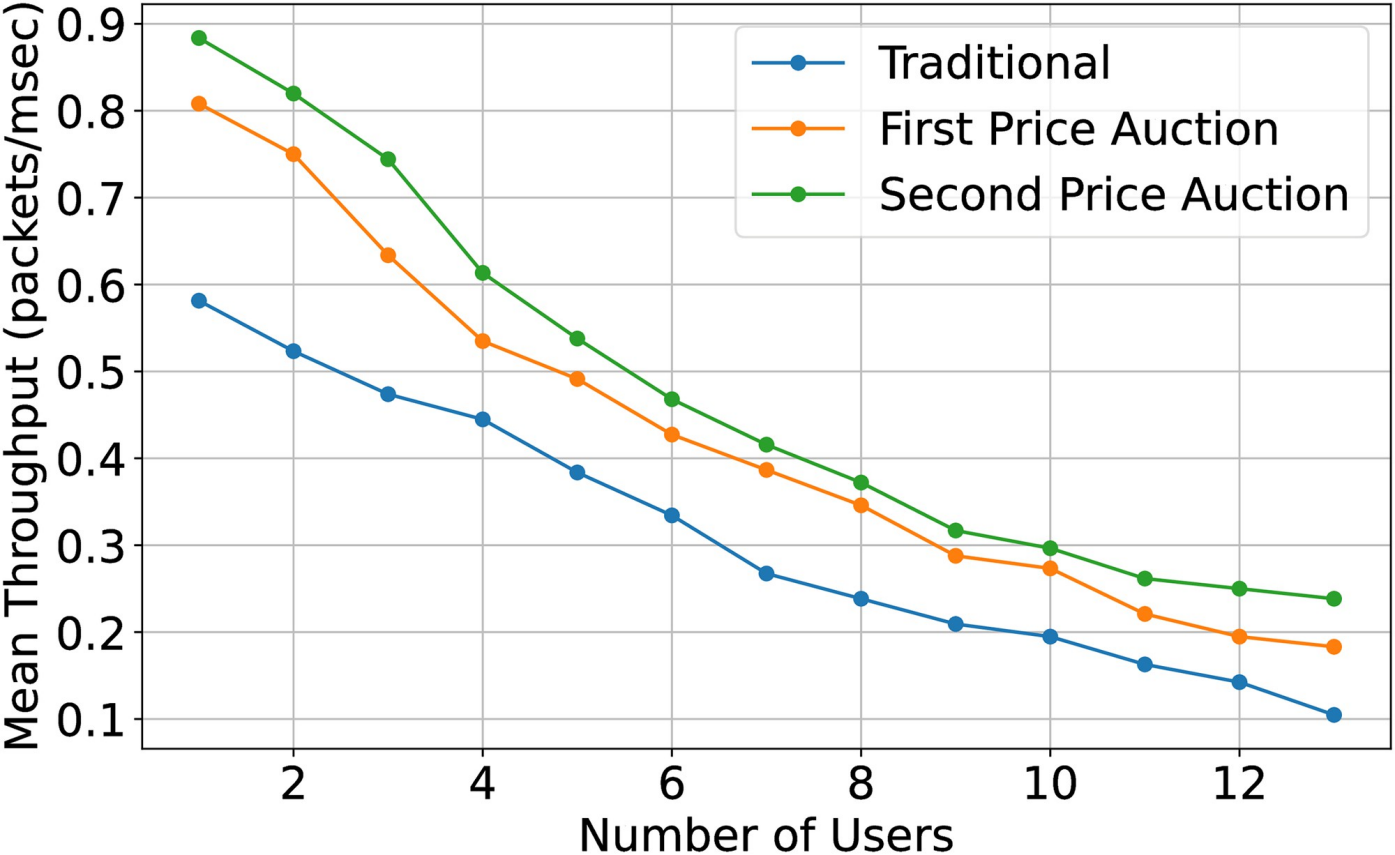
Mean throughput of three task distribution and sensing model.

The impact of the auction mechanism on the IoT node is seen the Fig 8 where the throughput of the IoT node is plotted against the number of IoT nodes. It is shown that the second price auction performs better than traditional sensing in terms of throughput for IoT nodes. This improvement can be attributed to the auction mechanism’s ability to incentivize IoT nodes to bid strategically based on their energy levels and packet deadlines. By adjusting their bid values accordingly, IoT nodes can optimize their participation in the sensing process, leading to improved throughput compared to traditional sensing methods where such optimization may not be possible.

### 4.1 Pre-analysis: Choice of specific auction mechanism

Relying on existing knowledge, a special optimal auction mechanism of the multi-item auction is still complex work. It is unnecessary for us and the cognitive node to find it. Furthermore, for the following reasons, we decide to use ‘The Second Price Sealed Auction’.

- The cognitive node need not take on risk from speculators.
- This model can effectively allocate resources regardless of whether the probability distribution density function of each node is the same.
- It can be an incentive to bid on its true valuation. And ease the thinking burden of sense nodes.
- Expected return in this model is larger than the ‘first-price sealed auction’.

Meanwhile, sense nodes can communicate with each other, so we need to prevent them from cooperating. This auction model can avoid bidding rings to some extent. Then we can consider every node to decide its bid independently.

The main disadvantage is that this model doesn’t maximize the income of the cognitive node. For example, if there are two bidders, one offers 100$, the other offers 1$, this model will cause the cognitive node to lose heavily.

But it doesn’t matter. Because in general, there are about 100 sense nodes every cognitive node has so that this scenario is extremely rare. And as one might say, on account of the dominant position of the cognitive node in the forwarding aspect, and also because sense nodes always need to forward data by cognitive, sense nodes are unlikely to have a lower evaluation and don’t want to miss any choice to get the cognitive node’s help. Besides, we can set a reserve price to avoid this situation (2.4.2).

So to sum up, Multiple ‘The Second Price Sealed Auction’ is a feasible choice.

### 4.2 Simulation and analysis

**Fig 9:**
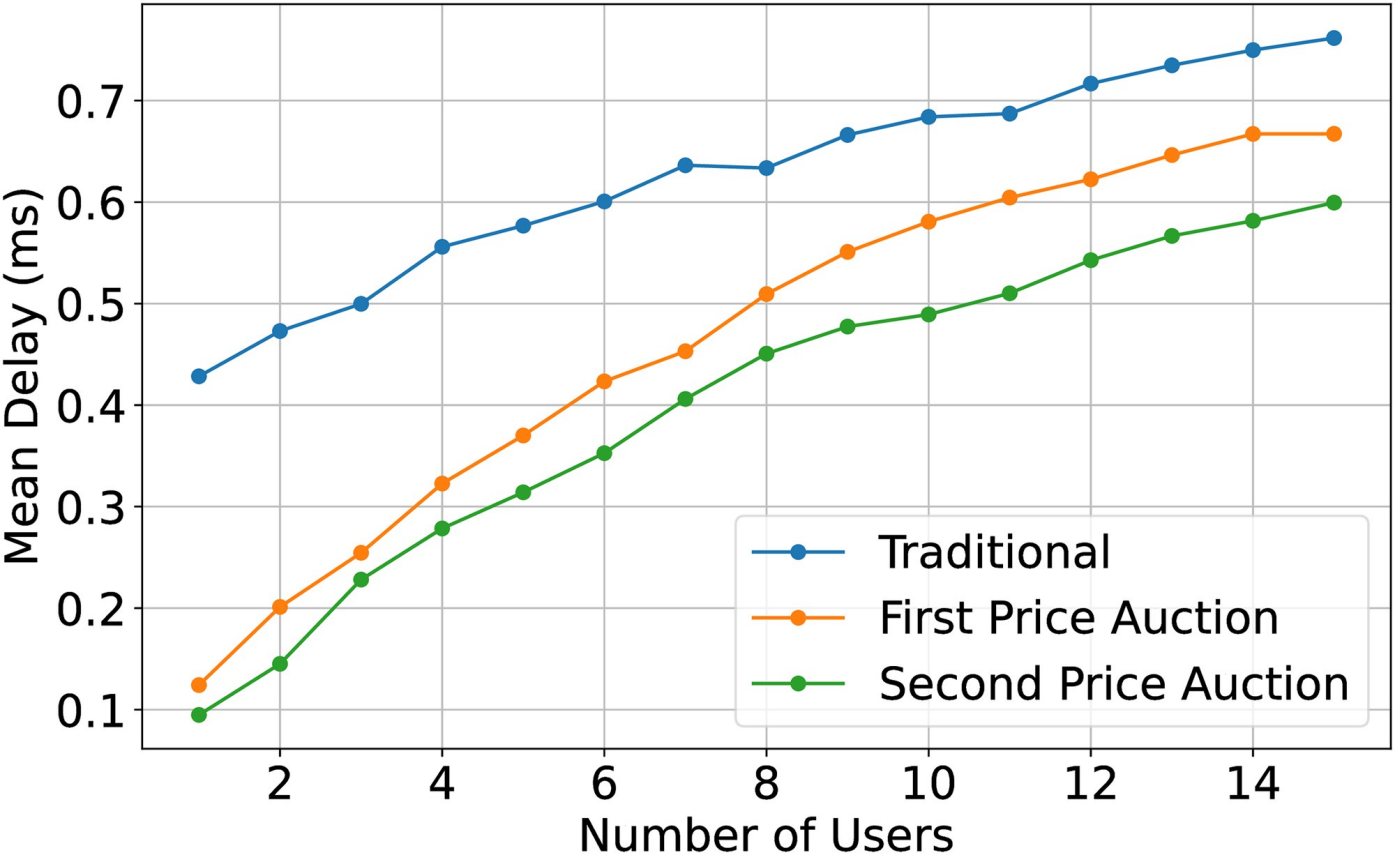
Mean delay with limit energy of three task distribution and sensing model.

**Fig 10:**
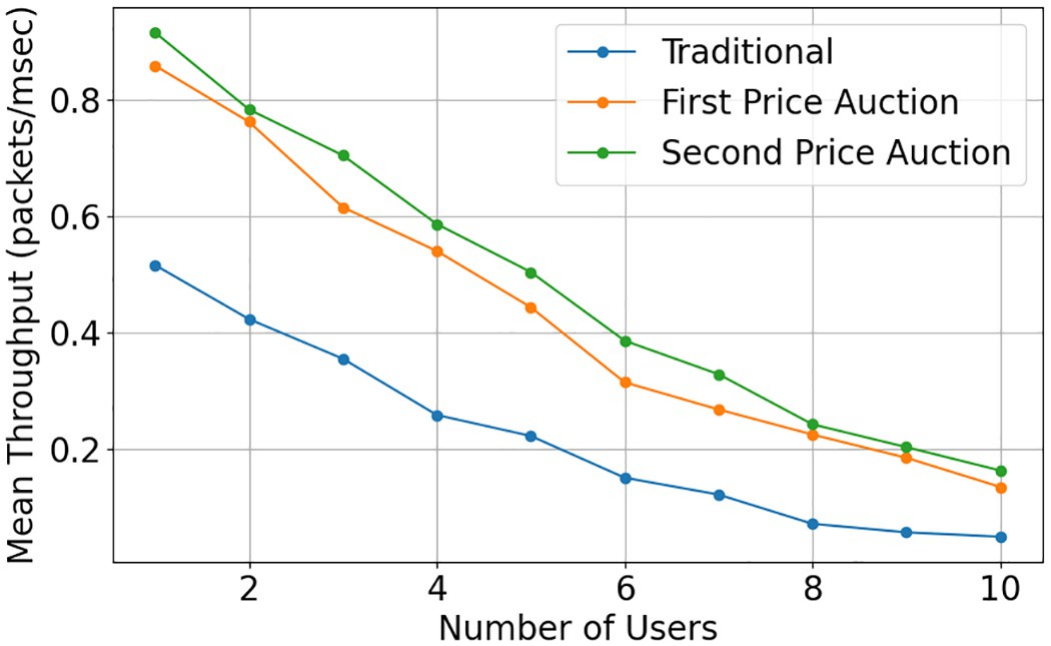
Mean throughput with limit energy and limit packet lifetime of three task distribution and sensing model.

In Figs 9 and 10, we study the performance of the CR using the first and second-price auctions. The performance enhancement, in this case, is the accurate out-of-band sensing by the IoT node. As explained in ([27]), the revenue generated by any auction is the same when the number of bidders exceeds 6. The proposed scheme enhances the performance through more reliable out-of-band sensing, reducing packet loss. The second price auction outperforms the first-price auction and in-band sensing. IoT nodes check their energy level and packet deadline and correspondingly adjust their bid value. The second price auction outperforms the first-price auction in these scenarios due to its ability to encourage IoT nodes to consider their energy levels and packet deadlines when placing bids. This consideration leads to more efficient resource utilization and task allocation, resulting in improved performance metrics such as reduced delay and enhanced throughput compared to traditional methods.

## 5 Conclusion

In this paper, a Punishment-Reward Strategy-based Cooperative approach for IoT Data Forwarding called PR-CSDF has been proposed. PR-CSDF consists of two parts: 1) Distribution of sensing task among the IoT nodes, 2) Enhancing the cooperation through reward and punishment strategy. Furthermore, we used the auction model to distribute the sensing task among the IoT nodes and to promote cooperation through the reward of licensed transmissions. Later, we used the optimal frequency of monitoring parameter to identify selfish and malicious nodes using DT and IDT. Finally, extensive simulations are conducted to show the benefits of PR-CSDF. Simulation results show that the PR-CSDF helps IoT nodes to effectively use their energy for sensing and data forwarding by reducing multiple re-transmissions through unlicensed channels.

The study showcases significant utility gains achieved by both secondary users and IoT nodes through the implementation of this strategy. By effectively optimizing cooperation, task allocation, and resource utilization via the punishment-reward mechanism, the approach not only enhances data forwarding efficiency but also improves energy efficiency within IoT networks. These results underscore the tangible benefits of integrating IoT technology with cooperative strategies in operations management, particularly in the context of smart city applications, where maximizing performance and resource efficiency are paramount for operational success.

## Supporting information

S1 Data (RAR)
